# Branched-chain and aromatic amino acid levels response to an oral glucose load associated with gestational diabetes mellitus

**DOI:** 10.1038/s41598-022-16539-y

**Published:** 2022-07-18

**Authors:** BeiBei Gao, Qiong Shen, Ying Wu, MengDie Cao, QiWu Zhang, Lei Chen

**Affiliations:** Department of Endocrinology, The Affiliated Suzhou Hospital of Nanjing Medical University, Suzhou Municipal Hospital, Suzhou, 215000 China

**Keywords:** Biomarkers, Diseases, Endocrinology, Medical research, Risk factors

## Abstract

Serum branched chain amino acids (BCAAs) and aromatic amino acids (AAAs) are associated with obesity, insulin resistance and type 2 diabetes mellitus (T2DM). We investigated the levels of these amino acids in women with gestational diabetes mellitus (GDM) and examined their changes in response to an oral glucose tolerance test (OGTT). 110 women were enrolled and underwent a 75-g OGTT during their second trimester; 43 women were diagnosed with GDM and 67 women did not have GDM (non-GDM women). During the OGTT, fasting, 1-h, and 2-h blood samples were obtained. BCAA and AAA levels were measured by liquid chromatography-tandem mass spectrometry. The differences in BCAA and AAA levels between GDM and non-GDM women were not evident during fasting but became significant after glucose loading. Glucose ingestion decreased the levels of BCAAs and AAAs in both groups. Notably, GDM women showed a delayed and blunted decrease in these amino acids compared to non-GDM women. The risks of 2-h changes in BCAAs and AAAs for GDM women were significant. We identified that the differences in BCAA and AAA levels between GDM women and controls, which were not evident during fasting, could be provoked by performing an OGTT.

## Introduction

Pregnancy is associated with remarkable changes in metabolism to support fetal demands and prepare for energy requirements in the postpartum period. In a healthy pregnancy, a decline in insulin sensitivity enhances the availability of metabolic substrates for the fetus^1,2^. However, women in whom insulin secretion does not increase appropriately to compensate for the increased insulin resistance are at high risk for gestational diabetes mellitus (GDM)^3^. GDM has increased dramatically in recent years and is defined as glucose intolerance observed during pregnancy^4,5^. GDM is associated with an increased risk of various maternal and perinatal complications, such as cesarean delivery, macrosomia, shoulder dystocia, neonatal hypoglycemia, hypocalcemia and subsequent type 2 diabetes mellitus (T2DM)^5,6^.

Metabolomics has provided novel approaches to understanding diabetes, thus attracting worldwide attention. Recently, a number of studies^7–9^ have shown that fasting levels of branched-chain amino acids (BCAAs) and aromatic amino acids (AAAs), including valine (Val), leucine (Leu), isoleucine (Ile), tyrosine (Tyr), and phenylalanine (Phe), are associated with obesity, insulin resistance, and T2DM in nonpregnant populations. In prospective studies, increased concentrations of fasting Val, Leu, Ile, Tyr, and Phe were associated with future diabetes^10^. Considering that T2DM and GDM share similar characteristics^11^, we hypothesized that these amino acids may be beneficial in studies regarding GDM. Some prior studies investigated the association between these amino acids and GDM; however, the results were relatively contradictory^12–14^.

Denise M. Scholtens and coworkers found that fasting Leu/Ile and Phe were higher and Val had a trend to be higher in mothers with high fasting blood glucose (FBG) levels than in mothers with low fasting blood glucose levels at ~ 28 weeks’ gestation^12^. Women with GDM had higher fasting Val, Leu, Ile, Tyr and Phe levels than women in the control group among obese pregnant women in their second trimester of pregnancy^13^. However, Danuta Dudzik et al. reported that there were no changes in fasting BCAA or AAA levels in women with GDM compared to women in the control group in the second trimester of pregnancy^14^.

Previous studies analyzing the differences in metabolite profiles between women with GDM and healthy controls mostly focused on the fasting state, which failed to reveal the metabolic changes and flexibility of the subjects. The oral glucose tolerance test (OGTT) is widely used as a standard method to establish the diagnosis of T2DM and provides a chance to observe the physiological changes during glucose ingestion^15,16^. An OGTT induces a transition from catabolism to anabolism, which is accompanied by many changes in metabolite concentrations, including those of BCAAs and AAAs, to achieve glucose homeostasis^17^. Studying the challenged metabolic state is important, as people in modern society spend most of the day in a postprandial state, and metabolic abnormalities may be masked in the fasting state.

To comprehensively understand the association between amino acids and GDM, we investigated the BCAA and AAA levels during an OGTT across 3 time points in women with GDM and healthy controls during the second trimester of pregnancy. This study may shed light on the metabolic dysregulation of BCAAs and AAAs underlying GDM, which is one of the most serious health problems today.

## Methods

### Study population

The study enrolled pregnant women aged 20–41 years at 20–29 weeks’ gestation who were receiving antenatal care at the Maternity and Child Health Center of Suzhou Municipal Hospital between August 1, 2017, and February 28, 2018. All subjects were of Han Chinese descent. Women with the following characteristics were excluded from the study: women with pre-existing diabetes mellitus; women with chronic diseases requiring medication during pregnancy, except for levothyroxine; women with a history of smoking; and women with missing data. Finally, 110 women were enrolled in the study and underwent a 75-g OGTT during their second trimester. According to the International Association of Diabetes and Pregnancy Study Groups criteria^15^, 43 women were diagnosed with GDM and 67 did not have GDM (non-GDM women).

All subjects provided written informed consent for inclusion in the study. Ethics approval was obtained from the hospital. This study was approved by the Research Ethics Committees of the Affiliated Suzhou Hospital of Nanjing Medical University and was carried out in accordance with the principles of the Declaration of Helsinki as revised in 2008.

### Characteristics collection

The anthropometric data [Pre-pregnancy weight (Pre-weight)], height, gestational age (weeks) at sample collection, lifestyle factors and history of medication were obtained from clinical medical records through a standard questionnaire. Systolic and diastolic blood pressure (expressed in mmHg) was measured twice (Omron Model HBP-1100, Omron Company, Dalian city, Liaoning Province, China) with the participant in a sitting position, and the mean value was used for further analysis. Pre-body mass index (Pre-BMI) was calculated as the subject’s Pre-weight (kg) divided by their height squared (m^2^).

### Samples and laboratory measurements

After an overnight fasting of at least 8 h, blood samples were drawn at three time points: fasting, 1-h (1-h), and 2-h (2-h) during the OGTT. Each time point had two blood samples. One was used for routine blood biochemical parameters by a fully automatic biochemical analyzer (Hitachi 7000, Tokyo, Japan) within one hour. Another was centrifugated and immediately frozen (− 80 °C) for detection of BCAAs and AAAs. Liquid chromatography-tandem mass spectrometry (LC–MS/MS) was used to quantify serum BCAAs and AAAs as we previously reported^18^. Chromatographic separation was carried out on a Syncronis HILIC column (150 mm × 2.1 mm, 5 μm, column temperature 35 °C; Shimadzu, Kyoto, Japan) with a mobile phase (acetonitrile:ammonium acetate = 89:11, v/v, 0.6 mL/min). The retention times for L-Val, L-Leu, L-Ileu, L-Phe and L-Tyr were 8.45 min, 5.33 min, 5.96 min, 4.56 min and 8.87 min, respectively. The MS analysis was performed using a QTrap 5500 mass spectrometer (AB Sciex, Concord, Ontario, Canada). Amino acid concentrations were determined using multiple reaction monitoring (MRM) with different transitions, a matrix effect of 98.7–107.3%, and a recovery of 92.7–102.3%^18^.

### Statistical analyses

The data were tested for normality (Shapiro–Wilk normality test) before analysis of variance. We showed Shapiro–Wilk normality test results for Characteristics in Table S1 and for amino acid concentrations in Table S2. We also used notched box-plots (Fig. S1) to show outliers of the data and did not find obvious outliers. All continuous variables except for gestational age could be processed according to the normal distribution; comparisons between the non-GDM and GDM groups were performed using Student’s t test except for gestational age. We used Mann–Whitney U test to compare gestational age. Within groups, we used a paired t test to compare amino acid values after the OGTT with fasting values. Percent changes of the amino acids at 1 h/2 h were calculated as follows: percent changes = (concentration at 1 h/2 h − concentration at fasting)/concentration at fasting × 100%. To analyze the change patterns between the two groups, a P < 0.05/5 = 0.01 was considered statistically significant after Bonferroni correction (5 amino acids). Binary logistic regression models were used to explore the association of BCAA and AAA levels with the risk of GDM. We used group category (GDM group and non-GDM group) as the response variable and fasting and 2-h changes in amino acid concentrations as the independent variables. 2-h change in amino acid concentrations were calculated as Concentration at 2-h − Concentration at fasting. Three models were used: (1) an unadjusted model; (2) a model adjusted for age and gestational age; and (3) a model adjusted for age, gestational age and pre-BMI. Results of logistic regression analysis were reported as odds ratio (OR) and 95% confidence interval(CI). Violin plot analyses were constructed using the Fluidigm Singular Analysis Toolset 3.5.2R package. Other statistical analyses were performed using the Statistical Package for the Social Sciences version 21.0 software, with a P < 0.05 (except for specially stated) considered significant. GraphPad Prism 5 was used to create the figures.

## Results

### Characteristics of the study population

The clinical characteristics of the 110 pregnant women at 20–29 weeks gestation are presented in Table 1. A total of 43 women with GDM (age, 30.98 ± 4.63 years) and 67 women without GDM (age, 29.10 ± 3.63 years) were included. Women with GDM were older and had a higher pre-BMI than the non-GDM women. Subjects in the GDM group had higher glucose levels at all three time points during the OGTT. There were also significant differences in SBP and DBP between the two groups.

**Table 1:** Characteristics of the study population.

	GDM (n = 43)	non-GDM (n = 67)	P
Age (years)	30.98 ± 4.63	29.10 ± 3.63	0.02
Pre-weight (kg)	70.93 ± 11.70	55.25 ± 8.04	< 0.001
Pre-BMI (kg/m^2^)	27.69 ± 4.76	21.33 ± 3.01	< 0.001
Gestational age (days)	170 (169–172)	181 (171–191)	< 0.001
SBP (mmHg)	120.40 ± 14.90	106 ± 11.95	< 0.001
DBP (mmHg)	75.4 ± 9.89	67.75 ± 8.48	< 0.001
HbA1c (%)	5.37 ± 0.55	4.77 ± 0.36	< 0.001
Fasting glucose (mmol/L)	5.53 ± 1.00	4.07 ± 0.40	< 0.001
1-h glucose (mmol/L)	11.64 ± 1.91	7.37 ± 1.31	< 0.001
2-h glucose (mmol/L)	9.43 ± 1.78	6.06 ± 1.08	< 0.001

Data were represented as mean ± standard deviation or median (interquartile range); Comparisons between the non-gestational diabetes mellitus (non-GDM) and the GDM groups were performed using student’s t-test except for gestational age. Comparison of gestational age between the non-GDM and the GDM groups was estimated by Mann–Whitney U test. The statistically significant difference was defined as P < 0.05.

*Pre-weight* pre-pregnancy weight, *Pre-BMI* pre-body mass index, *SBP* systolic blood pressure, *DBP* diastolic blood pressure, *HbA1c* hemoglobin A1c, *OGTT* oral glucose tolerance test, *1-h* *glucose* one-hour glucose at OGTT, *2-h* *glucose*, two-hour glucose at OGTT.

### Comparison of serum amino acid concentrations during an oral glucose tolerance test between women with gestational diabetes mellitus (GDM) and women without GDM (non-GDM)

We examined the amino acid concentrations in each group (Table 2). There was no significant difference between the GDM group and non-GDM group in fasting status. After a 75-g OGTT, amino acid levels differed between the GDM group and the non-GDM group. All of the 1-h amino acid levels in the GDM group, including Val, Leu, Ile, Tyr, and Phe levels, were higher than those in the non-GDM group. Moreover, except for Phe, the amino acid levels at 2 h showed similar results to those at 1 h. To show data distribution and original values, the results based on the groups were graphically illustrated in Fig. S2 using violin plots.

**Table 2:** Comparisons of serum amino acid concentrations in the non-gestational diabetes mellitus (non-GDM) and GDM during an oral glucose tolerance test (OGTT).

Amino acids (µmol/L)	GDM (n = 43)	non-GDM (n = 67)	P
0-Val	199.62 ± 33.15	201.8 ± 24.69	0.694
1-h Val	200.32 ± 32.33	170.14 ± 22.74	< 0.001
2-h Val	166.65 ± 27.86	151.31 ± 20.92	0.001
0-Leu	119.91 ± 20.64	120.70 ± 14.02	0.810
1-h Leu	118.30 ± 22.33	92.52 ± 12.12	< 0.001
2-h Leu	89.56 ± 16.21	77.91 ± 11.76	< 0.001
0-Ile	60.93 ± 9.91	59.04 ± 7.81	0.266
1-h Ile	62.04 ± 14.53	42.37 ± 6.85	< 0.001
2-h Ile	45.04 ± 10.64	33.10 ± 6.25	< 0.001
0-Tyr	42.16 ± 7.40	44.22 ± 6.42	0.125
1-h Tyr	46.73 ± 8.16	35.85 ± 5.89	< 0.001
2-h Tyr	38.52 ± 7.60	29.42 ± 5.34	< 0.001
0-Phe	91.39 ± 19.29	91.17 ± 12.96	0.947
1-h Phe	87.72 ± 14.20	80.30 ± 12.20	0.004
2-h Phe	78.53 ± 14.16	74.53 ± 11.37	0.105

Values were expressed as mean ± standard deviation. Comparisons between the non-gestational diabetes mellitus (non-GDM) and GDM groups were performed using student’s t-test. The statistically significant difference was defined as P < 0.05.

*Val* valine, *Leu* leucine, *Ile* isoleucine, *Tyr* tyrosine, *Phe* phenylalanine, *0* fasting, *1-h* one-hour at OGTT, *2-h* two-hour at OGTT.

### Changes in amino acids during the oral glucose tolerance test (OGTT)

**Figure 1:**
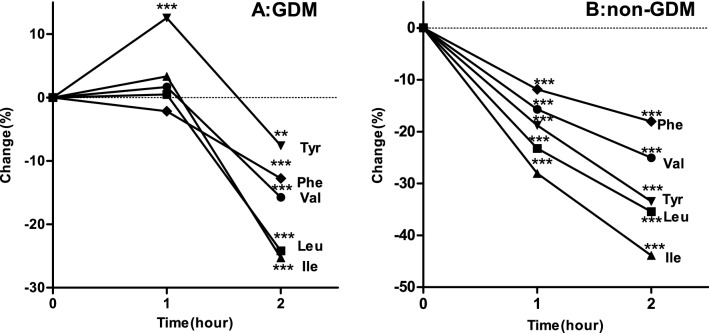
Amino acid changes in response to an oral glucose tolerance test. The dots denoted mean percent change. Percent change was defined as the absolute change in relative to baseline. (**A**) Women with gestational diabetes mellitus (GDM) group. (**B)** Non-GDM group. *P < 0.05, **p < 0.01, ***P < 0.001, compared amino acids values at corresponding time point after glucose ingestion with fasting using the paired t test.

**Figure 2:**
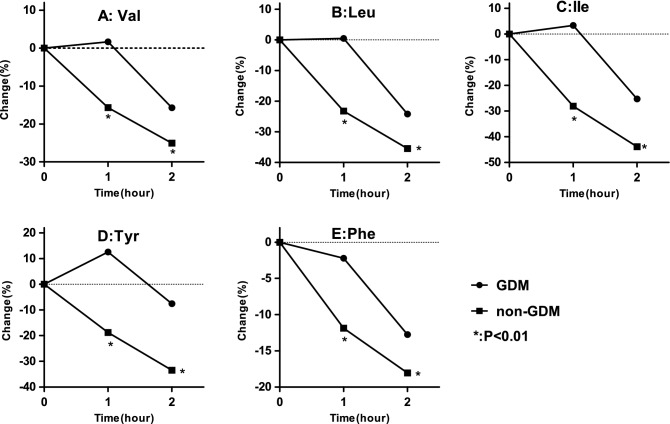
Percent changes of amino acid between the gestational diabetes mellitus (GDM) and non-GDM groups. The dots denoted mean percent change. Percent change was defined as the absolute change in relative to baseline. (**A**) Val. (**B**) Leu. (**C**) Ile. (**D**) Tyr. E Phe. *P < 0.01, compared percent changes of amino acid at corresponding time point between GDM and non-GDM using student’s t test.

The changes in amino acids during the OGTT in the GDM group and non-GDM group are illustrated in Fig. S3, Figs. 1 and 2. Compared with the baseline levels, all amino acid levels at 2 h were significantly reduced after the OGTT in both groups (Fig. S3A,B). We also described the percent changes and the significance of the changes in both groups (Fig. 1A,B), and specific results are available in Table S3. In the GDM group, compared to the baseline levels, the Val, Leu, Ile and Phe levels were unaffected at 1 h and were reduced significantly at 2 h, ranging from 12.75 to 25.30%. The Tyr levels primarily increased by 12.56% at 1 h and decreased by 7.59% at 2 h. In the non-GDM group, compared to baseline, all amino acid levels displayed a small decrease at 1 h, which became more pronounced at 2 h. On the other hand, our results revealed that the percent change in Ile was 43.90% in the non-GDM group, which was largest change among the amino acids. Similarly, the largest percent change in the GDM group was also for Ile. Furthermore, we compared the percent changes in the two groups (Fig. 2), and specific results are shown in Table S4. Pronounced differences were observed after glucose ingestion between the two groups. Val, Leu, Ile and Phe showed a delayed decrease in the GDM compared to the non-GDM group; within the first hour, there was no significant decrease in Val, Leu, Ile and Phe in the GDM group. At 2 h, the GDM group displayed a smaller decrease in all five amino acid levels than the non-GDM group.

### The odds ratio (OR) for the association of amino acids with gestational diabetes mellitus (GDM)

A logistic regression model was used to analyze the risks of changes in amino acid levels (fasting and 2-h changes in amino acids) for GDM (Table 3). In the crude model, the risks of fasting BCAA levels (Val, Leu, and Ile) and fasting AAA levels (Tyr and Phe) for GDM were insignificant. We further discovered that the 2-h changes in BCAA and AAA levels for GDM were evidently significant. These significant associations remained the same or became slightly attenuated after adjustment for age and gestational age. In contrast, the associations were moderately attenuated after further adjusting for Pre-BMI. The OR of the 2-h change in Val, Ile, and Tyr levels for GDM still existed, while the 2-h response of Leu and Phe levels disappeared.

**Table 3:** The cruel and adjusted logistic regression model of amino acids with gestational diabetes mellitus.

	Fasting			2 h-change		
	Beta (95% CI)	Beta (95% CI)	Beta (95% CI)	Beta (95% CI)	Beta (95% CI)	Beta (95% CI)
	Model 1	Model 2	Model 3	Model 1	Model 2	Model.3
Val	0.997 (0.984,1.011)	1.001 (0.985,1.017)	0.991 (0.971,1.010)	1.061 (1.031,1.092)***	1.060 (1.027,1.093)***	1.035 (1.001,1.070)*
Leu	0.997 (0.975,1.020)	1.002 (0.974,1.030)	0.985 (0.952,1.018)	1.070 (1.034,1.106)***	1.071 (1.029,1.115)**	1.040 (0.996,1.085)
Ile	1.026 (0.981,1.073)	1.019 (0.966,1.075)	0.970 (0.906,1.038)	1.149 (1.084,1.218)***	1.157 (1.082,1.238)***	1.108 (1.028,1.194)**
Tyr	0.955 (0.901,1.013)	0.952 (0.891,1.018)	0.942 (0.867,1.024)	1.445 (1.255,1.665)***	1.423 (1.227,1.650)***	1.302 (1.118,1.516)**
Phe	1.001 (0.977,1.026)	1.000 (0.972,1.028)	0.990 (0.957,1.024)	1.044 (1.001,1.089)**	1.044 (0.996,1.095)	1.017 (0.964,1.073)

Results are based on analyses of the study population [43 women with gestational diabetes mellitus (GDM) and 67 women without GDM (non-GDM)].

Values represent regression coefficients from the logistic regression analyses and their 95% confidence intervals (C.I.) for the risk of GDM.

The statistically significant difference was defined as P < 0.05. *P < 0.05, **P < 0.01, ***P < 0.001.

2-h changes which were calculated as Concentration at 2-h-Concentration at fasting.

Model 1: unadjusted. Model 2: adjusted for age and gestational age. Model 3: adjusted for age, gestational age and Pre-BMI.

## Discussion

In this study, we profiled three time points of BCAA and AAA levels during a 75-g OGTT in 43 women with GDM and 67 healthy controls during their second trimester using a targeted metabolomics approach. Overall, the levels of the BCAAs and AAAs did not differ significantly between the GDM and non-GDM groups in the fasting status. After glucose loading, our data on the 2-h levels of BCAAs and AAAs apparently decreased compared to the fasting status in both groups, supporting the known action of insulin in the suppression of proteolysis. Furthermore, we found different patterns of change in BCAA and AAA levels after glucose loading between the two groups, with a delayed and blunted decrease in GDM. We also observed negative relationships between 2-h changes in BCAA and AAA levels and GDM.

According to previous studies, increasing levels of fasting Val, Leu, Ile, Tyr, and Phe are most consistently associated with obesity and T2DM outside of pregnancy^7–9^. However, the associations between these amino acids and GDM are inconsistent. Fasting Leu/Ile and Phe levels were higher, and Val levels had a trend to be higher in Northern European mothers between 24 and 32 weeks’ gestation with high fasting blood glucose (FBG) levels (> 90th percentile) than in mothers with low FBG levels (< 10th percentile)^12^. Similarly, Sara L. White performed a metabolic study among obese pregnant women in their second trimester of pregnancy and found that women with GDM had higher fasting Val, Leu, Ile, Tyr, and Phe levels than women in the control group^13^. In contrast to the former two studies, our study did not find any differences in fasting BCAA and AAA levels between the GDM and non-GDM groups. This finding was in agreement with Danuta Dudzik et al., who compared the metabolic profiles between women with GDM and women in the control group during their second trimester using targeted metabolomics^14^. The discrepancy could be explained by the differences in races, ages, maternal BMI, methods of assessing amino acid levels and small sample sizes. One recent study including 83 pregnant women at ≥ 25 weeks’ gestation showed significantly elevated levels of Val, Ile, Tyr and Phe in pregnant women with T2DM but not in mothers with GDM^19^. Another study from America investigating the effects of GDM on intermediary metabolism in late pregnancy observed that women with GDM whose fasting glucose level was 5.83 mmol/L or greater had elevated levels of fasting Val, Leu, and Ile compared with controls^20^. Meanwhile, they did not observe any differences between subjects with GDM whose fasting glucose was less than 5.83 mmol/L and controls. Combining the latter two studies, we speculated that different glucose metabolism disorders with varying degrees of islet dysfunction might be another reason for the discrepancy.

An earlier report showed a gradual decrease in Val, Leu, Ile, Tyr, and Phe levels after glucose ingestion in nonpregnant populations with normal glucose tolerance, which was in agreement with the population of healthy pregnant women in this study^21^. Notably, we found marked differences between healthy controls and women with GDM in glucose-provoked alterations in these amino acids. Val, Leu, Ile and Phe levels remained unchanged, and Tyr levels increased during the first hour of the OGTT in the GDM group, after which they showed a blunted decrease. A previous study showed that Val, Leu, Ile and Tyr levels increased during the first 30 min of the OGTT and then showed a blunted decrease in obese individuals^22^. The patterns of decreases (delayed and blunted) in these amino acids in the GDM group, similar to those in obese individuals, reflected a dysregulation of proteolysis, which might be partly associated with impaired insulin sensitivity. Furthermore, we noticed that the percent changes in the amino acids at 2 h in healthy pregnant women were smaller in comparison to those found by Qin Wang in nonpregnant healthy populations ^21^. However, the percent changes were similar in extent to insulin-resistant individuals and those with prediabetes. Additionally, we observed that percent changes at 2 h in GDM patients generally decreased less than those in healthy controls and were even less than those in the T2DM patients in Qin Wang’s study^21^. We hypothesized that insulin resistance in women with GDM during the second trimester of pregnancy seemed to be more severe than that in individuals with T2DM.

In our study, the risks of 2-h changes in BCAA and AAA levels for GDM were significant, while fasting BCAA and AAA levels were not significant. According to a previous study, linear regression models revealed that 2-h changes in leucine/isoleucine and valine levels were associated with fasting insulin levels in individuals with impaired glucose tolerance^23^. Furthermore, another study from Sweden including 21 healthy individuals discovered that the postprandial responses of the three diabetes-associated amino acids (DMAAs, Ile, Tyr, and Phe) were more strongly associated with fasting glucose levels than the fasting concentrations of the DMAAs^24^. Mook-Kanamori et al. also found more associations among postprandial amino acid concentrations than in the fasting state in T2DM^25^. We showed that the physiological challenges increased interindividual variation, revealing metabotypes that were not evident at baseline. The link between the 2-h change in BCAA and AAA levels and GDM remained the same or became slightly attenuated after adjustment for age and gestational age, while it was moderately attenuated after further adjustment for Pre-BMI. This decrease suggested that Pre-BMI accounted in part for the risks of changes in BCAA and AAA levels for GDM.

Investigation of BCAA and AAA levels in response to an oral glucose test gave us a unique opportunity to study the changes from catabolism to anabolism. After the OGTT, the differences remained consistent regardless of whether the metabolic changes were assessed via relative or absolute concentration changes. Our study revealed that the postprandial dysfunction of BCAAs and AAAs might be a novel component of GDM that has hitherto been poorly recognized as a potential interventional target. However, our study still has limitations. Due to its small sample size, confirmation studies should include larger groups. We merely investigated the concentrations of BCAAs and AAAs at one time point during the second trimester. Since metabolism changes substantially in the maternal body in different trimesters of pregnancy, it is important to explore longitudinal metabolomic profiles across gestation in the future.

In conclusion, our results suggested that the differences in BCAA and AAA levels between women with GDM and controls during their second trimester, which were not evident during fasting, could be determined by performing an OGTT. The different patterns of decreases in BCAA and AAA levels in GDM patients and controls provided a deeper understanding of the GDM phenotype. It might therefore be beneficial to better understand postprandial dysfunction in GDM beyond fasting. We maintained that this was particularly important because people mostly remain in a non-fasting state and because it might provide new therapeutic opportunities.

## Supplementary Information

Supplementary Information.

## Data Availability

The datasets generated during and analysed in the current study are available from the corresponding author on reasonable request.
