# Supplementary material for: Branched-chain and aromatic amino acid levels response to an oral glucose load associated with gestational diabetes mellitus
Source: Sci Rep. 2022 Jul 18;12:12257. doi: 10.1038/s41598-022-16539-y (PMC9293928; doi:10.1038/s41598-022-16539-y)
Supplement: Supplementary file 1 — Supplementary Information. [file 41598_2022_16539_MOESM1_ESM.pdf]

Branched-chain and aromatic amino acid levels response to an oral glucose load associated with gestational diabetes mellitus

BeiBei GAO[#], Qiong SHEN[#], Ying WU, MengDie CAO, QiWu ZHANG, Lei CHEN[*]

Department of Endocrinology, The Affiliated Suzhou Hospital of Nanjing Medical University, Suzhou Municipal Hospital, Suzhou 215000, China

[#]These authors contributed equally to this work.

[*]Corresponding author

Lei Chen, Department of Endocrinology, The Affiliated Suzhou Hospital of Nanjing Medical University, Suzhou Municipal Hospital, No. 126 Daoqian Street, Suzhou 215000, China

Email: szslyynfm@163.com

Table S1. shapiro-Wilk normality test for Characteristics

	Group	W	P
Age	non-GDM	0.965119	0.057
	GDM	0.981468	0.7053
Pre-weight	non-GDM	0.974723	0.1889
	GDM	0.985748	0.8632
Pre-BMI	non-GDM	0.9671	0.073
	GDM	0.943606	0.035
Gestational age	non-GDM	0.678286	<0.0001
	GDM	0.919639	0.0052
SBP	non-GDM	0.947661	0.0069
	GDM	0.963782	0.1904
DBP	non-GDM	0.894445	<0.0001
	GDM	0.970166	0.3204
HBA1c	non-GDM	0.984047	0.5464
	GDM	0.928284	0.0102
Fasting glucose	non-GDM	0.990394	0.8882
	GDM	0.95839	0.121
1-h glucose	non-GDM	0.973245	0.1574
	GDM	0.954238	0.0852
2-h glucose	non-GDM	0.982219	0.4527
	GDM	0.981961	0.7247

Through the above data analysis, we found that the indicators conformed to the normal distribution, except for Pre-BMI (GDM), Gestational age (non-GDM and GDM), SBP (non-GDM), DBP (non-GDM), HBA1c (GDM). Pre-BMI (GDM), SBP (non-GDM), DBP (non-GDM), HBA1c (GDM) could be treated as normal distribution according to other articles [1,2] and other groups. Gestational age was processed according to the non-normal data and the Mann-Whitney U test was carried out.

Pre-weight, Pre-pregnancy weight; Pre-BMI, Pre-body mass index; SBP, systolic blood pressure; DBP, diastolic blood pressure; HbA1c, hemoglobin A1c; OGTT, oral glucose tolerance test; 1-h glucose, one-hour glucose at OGTT; 2-h glucose, two-hour glucose at OGTT.

TableS2. shapiro-Wilk normality test for amino acids

	Group	W	P
0-Val	non-GDM	0.969442	0.0979
	GDM	0.984056	0.8045
1-h Val	non-GDM	0.956822	0.0205
	GDM	0.983052	0.7669
2-h Val	non-GDM	0.965467	0.0595
	GDM	0.975431	0.4776
0-Leu	non-GDM	0.978109	0.2844
	GDM	0.982008	0.7265
1-h Leu	non-GDM	0.981557	0.4215
	GDM	0.967595	0.2606
2-h Leu	non-GDM	0.981776	0.4316
	GDM	0.971049	0.3434
0-Ile	non-GDM	0.971352	0.1244
	GDM	0.969906	0.3138
1-h Ile	non-GDM	0.981783	0.432
	GDM	0.91677	0.0042
2-h Ile	GDM	0.978202	0.2875
	non-GDM	0.977374	0.5469
0-Tyr	GDM	0.952857	0.0127
	non-GDM	0.926889	0.0091
1-h Tyr	non-GDM	0.972076	0.1361
	GDM	0.983574	0.7866
2-h Tyr	non-GDM	0.967076	0.0728
	GDM	0.985437	0.8529
0-Phe	non-GDM	0.975063	0.197
	GDM	0.976454	0.5134
1-h Phe	non-GDM	0.982204	0.452
	GDM	0.960228	0.1413
2-h Phe	non-GDM	0.989968	0.8692
	GDM	0.966543	0.2392

Through the above data analysis, we found that the indicators conformed to the normal distribution, except for 0-Val (non-GDM), 1-h Ile (GDM) and 0-Tyr (non-GDM and GDM). Combined with the analysis of other articles[1,2] and the data in other time periods which belonged to the normal distribution, so it could be treated as normal distribution.

Val, valine; Leu, leucine; Ile, isoleucine; Tyr, tyrosine; Phe, phenylalanine; 0, fasting; 1-h, one- hour at OGTT; 2-h, two-hour at OGTT

Table S3. The percent changes of amino acids and significance of changes in both groups

Group	Amino acids	Time point	Mean percent	95%CI - lower	95%CI - higher	Pvalue
GDM	Val	1-h	1.66341	-3.36695	6.69376	0.508
GDM	Val	2-h	-15.75283	-19.33409	-12.17158	<0.001
GDM	Leu	1-h	0.47685	-5.86873	6.82243	0.88
GDM	Leu	2-h	-24.2067	-28.25792	-20.15547	<0.001
GDM	Ile	1-h	3.33744	-4.43348	11.10837	0.391
GDM	Ile	2-h	-25.29719	-30.46335	-20.13103	<0.001
GDM	Tyr	1-h	12.55069	6.00318	19.09821	<0.001
GDM	Tyr	2-h	-7.58852	-12.91995	-2.25709	0.006
GDM	Phe	1-h	-2.1599	-6.57951	2.25971	0.33
GDM	Phe	2-h	-12.74936	-16.47967	-9.01904	<0.001
non-GDM	Val	1-h	-15.7101	-16.67572	-14.74447	<0.001
non-GDM	Val	2-h	-25.04481	-26.11353	-23.9761	<0.001
non-GDM	Leu	1-h	-23.24527	-24.69939	-21.79116	<0.001
non-GDM	Leu	2-h	-35.44708	-37.02718	-33.86698	<0.001
non-GDM	Ile	1-h	-28.08552	-30.07824	-26.09279	<0.001
non-GDM	Ile	2-h	-43.89622	-45.90503	-41.88741	<0.001
non-GDM	Tyr	1-h	-18.78683	-20.64676	-16.92689	<0.001
non-GDM	Tyr	2-h	-33.47002	-35.14018	-31.79986	<0.001
non-GDM	Phe	1-h	-11.86623	-13.29597	-10.4365	<0.001
non-GDM	Phe	2-h	-18.03612	-20.00259	-16.06966	<0.001

Val, valine; Leu, leucine; Ile, isoleucine; Tyr, tyrosine; Phe, phenylalanine; 1-h, one- hour at OGTT; 2-h, two-hour at OGTT

Table S4. Differences in the percent changes of amino acids between GDMs and non-GDMs

Group	Amino acids	Time point	Mean percent change in each group	Mean difference in the percent changes betwwen two groups	95%CI - lower	95%CI - higher	Pvalue
GDM	Val	1-h	1.6634	-17.3735	-22.487	-12.26	<0.001
non-GDM	Val	1-h	-15.7101				
GDM	Val	2-h	-15.7528	-9.29198	-13.015	-5.5685	<0.001
non-GDM	Val	2-h	-25.0448				
GDM	Ile	1-h	0.4769	-23.72213	-30.217	-17.2273	<0.001
non-GDM	Ile	1-h	-23.2453				
GDM	Ile	2-h	-24.2067	-11.24038	-15.564	-6.91638	<0.001
non-GDM	Ile	2-h	-35.4471				
GDM	Leu	1-h	-28.0855	-31.42296	-39.422	-23.4236	<0.001
non-GDM	Leu	1-h	3.3374				
GDM	Leu	2-h	-25.2972	-18.59903	-24.111	-13.0871	<0.001
non-GDM	Leu	2-h	-43.8962				
GDM	Tyr	1-h	-18.7868	-31.33752	-38.121	-24.5542	<0.001
non-GDM	Tyr	1-h	12.5507				
GDM	Tyr	2-h	-33.47	-25.8815	-31.446	-20.317	<0.001
non-GDM	Tyr	2-h	-7.5885				
GDM	Phe	1-h	-11.8662	-9.70634	-14.332	-5.08084	<0.001
non-GDM	Phe	1-h	-2.1599				
GDM	Phe	2-h	-18.0361	-5.28677	-9.4687	-1.10485	0.14
non-GDM	Phe	2-h	-12.7494				

Val, valine; Leu, leucine; Ile, isoleucine; Tyr, tyrosine; Phe, phenylalanine; 1-h, one- hour at OGTT; 2-h, two-hour at OGTT

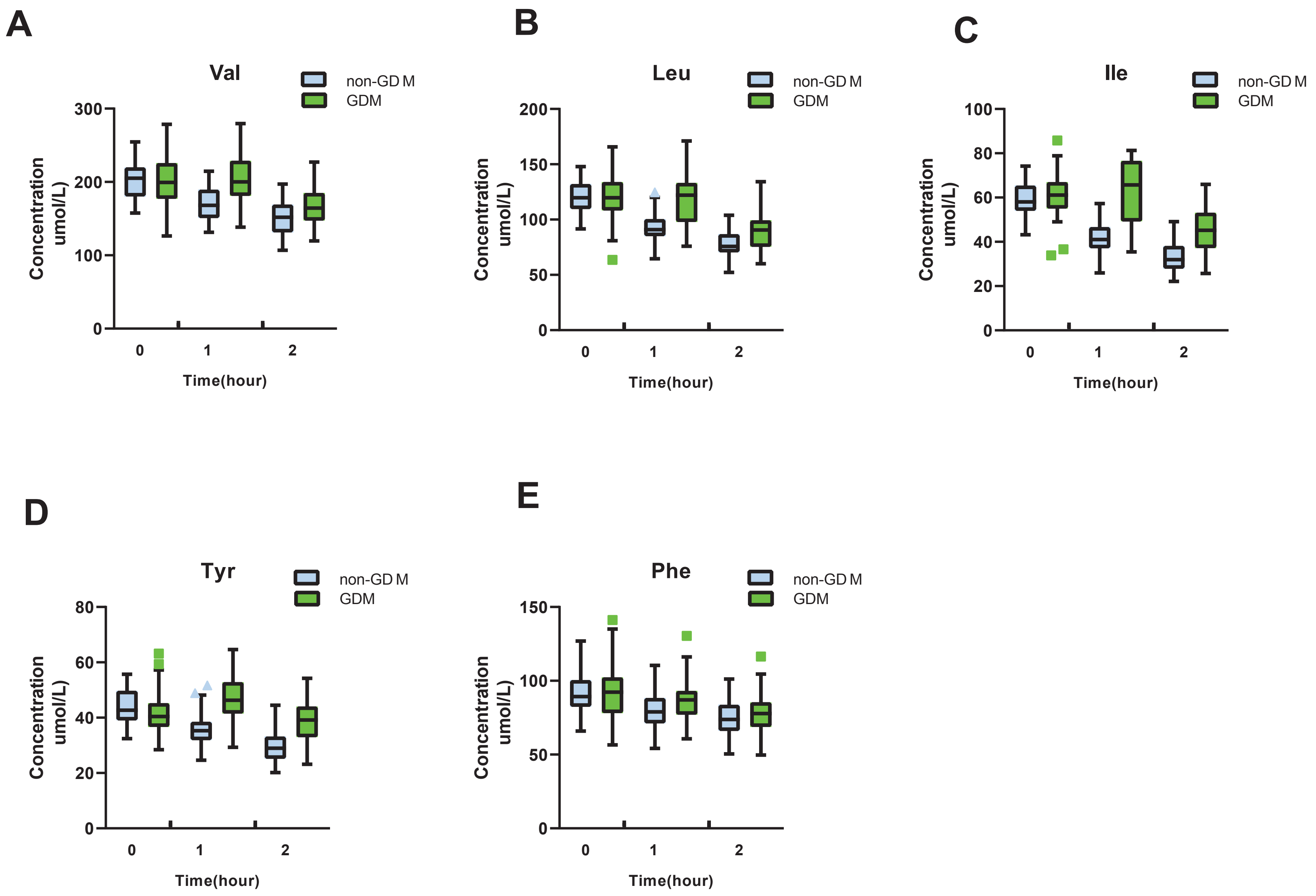

Fig. S1 Notched box-plots showing the distribution and outliers of the data in gestational diabetes mellitus (GDM) and non-GDM groups. A Val, valine; B Leu, leucine; C Ile, isoleucine; D Tyr, tyrosine; E Phe, phenylalanine.

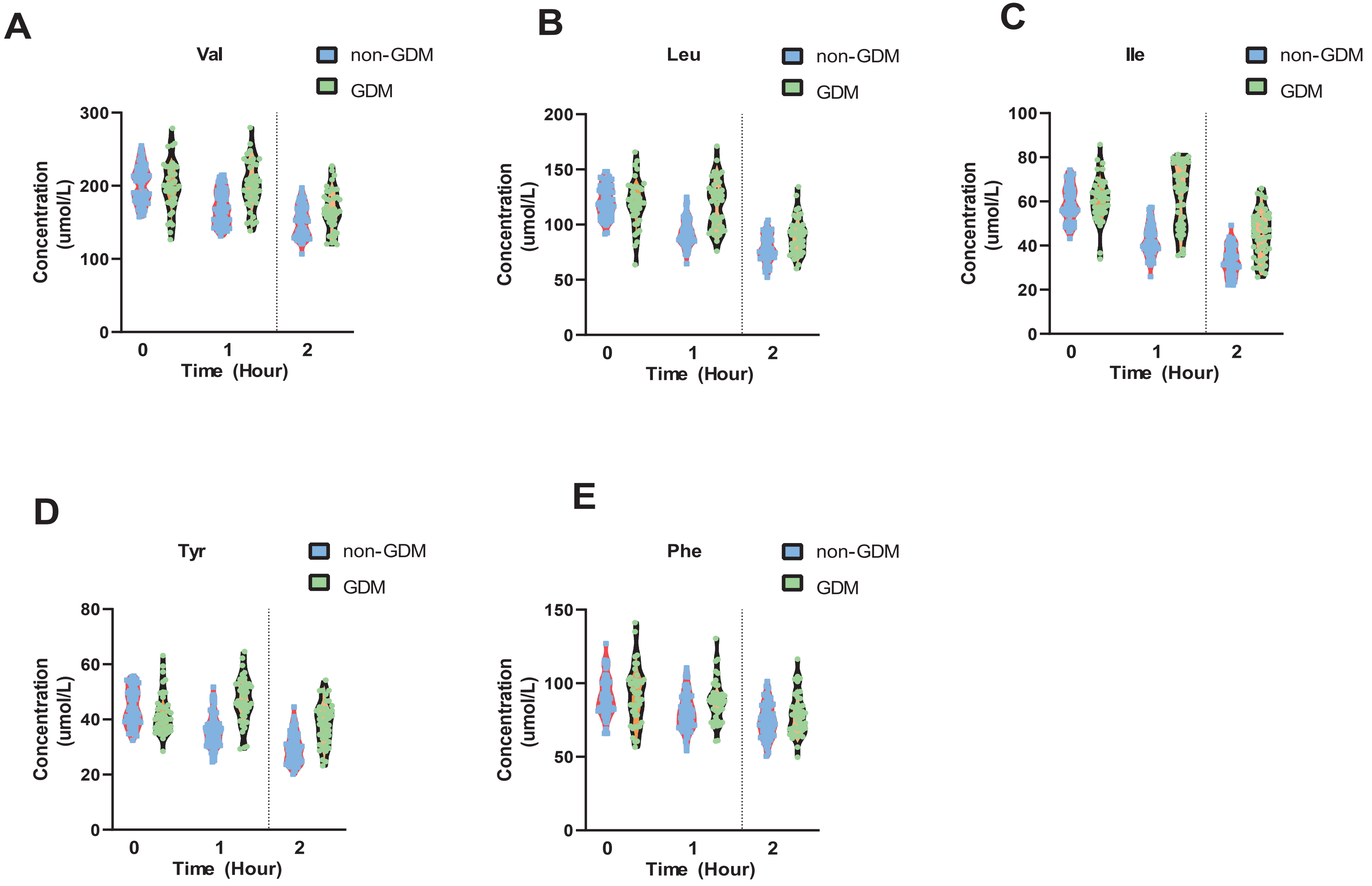

Fig. S2 Violin plot analyses comparing the levels and distributions of branched-chain （BCAAs） and aromatic amino acids （AAAs） in gestational diabetes mellitus (GDM) and non-GDM groups.

A Val, valine; B Leu, leucine; C Ile, isoleucine; D Tyr, tyrosine; E Phe, phenylalanine.

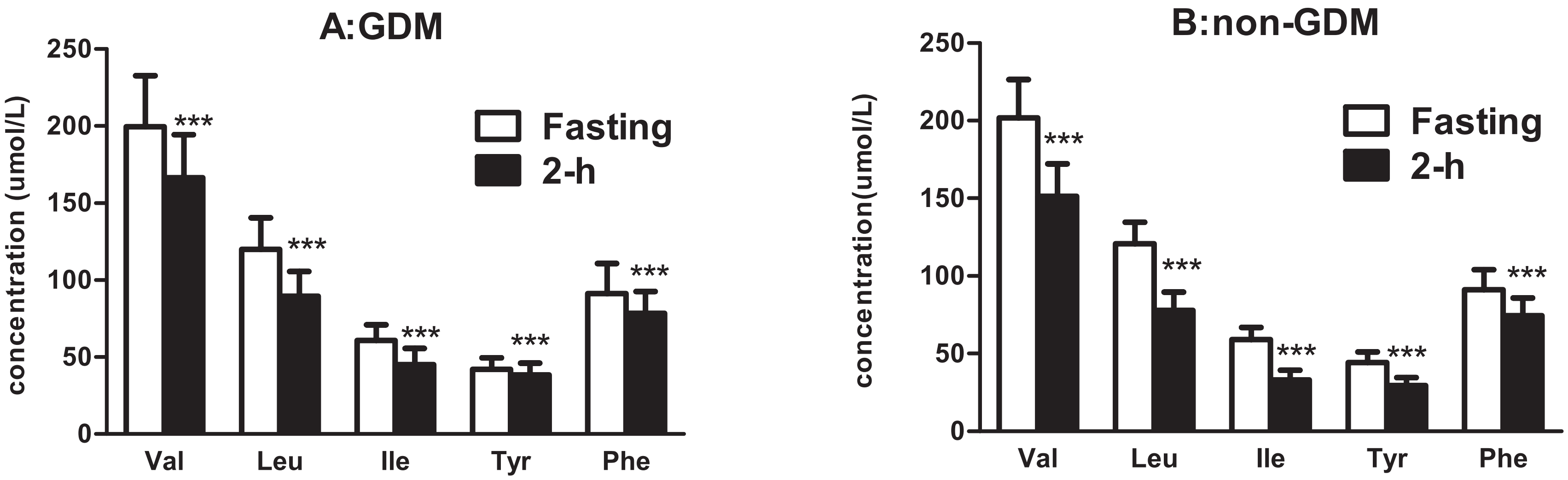

Fig. S3 Concentrations of BCAAs and AAAs (Mean ± SD) between fastinng and 2-h after glucose ingestion.
A women with gestational diabetes mellitus(GDM) group. B non-GDM group.
* $p < 0.05$, ** $p < 0.01$, *** $p < 0.001$, compared amino acids values at 2-h after glucose ingestion with fasting using the paired t test.

References

[1] Li Q.*et al*. Amino Acid and Biogenic Amine Profile Deviations in an Oral Glucose Tolerance Test: A Comparison between Healthy and Hyperlipidaemia Individuals Based on Targeted Metabolomics. *Nutrients* **8**, 379, https: // doi: 10.3390/nu8060379 (2016).

[2] Yang P. *et a*l. The positive association of branched-chain amino acids and metabolic dyslipidemia in Chinese Han population. *Lipids Health Dis***15**, 120, https: // doi: 10.1186/s12944-016-0291-7 (2016).
